# Presidential address 2017 William Harkness FRCS October 10th 2017 Denver, Co USA: 2017—annus mirabilis, a global view of neurosurgery for children

**DOI:** 10.1007/s00381-018-3931-6

**Published:** 2018-08-18

**Authors:** William F. J. Harkness

**Affiliations:** Great Ormond Street Hospital For Children NHS Trust, London, UK

**Keywords:** Global neurosurgery for children, Lancet commission, Global initiative for children’s surgery, Advocacy and lobbying

## Abstract

The following presidential address was delivered at the 45th Annual Meeting of the ISPN held in Denver, CO, USA in October 2017.

**Electronic supplementary material:**

The online version of this article (10.1007/s00381-018-3931-6) contains supplementary material, which is available to authorized users.

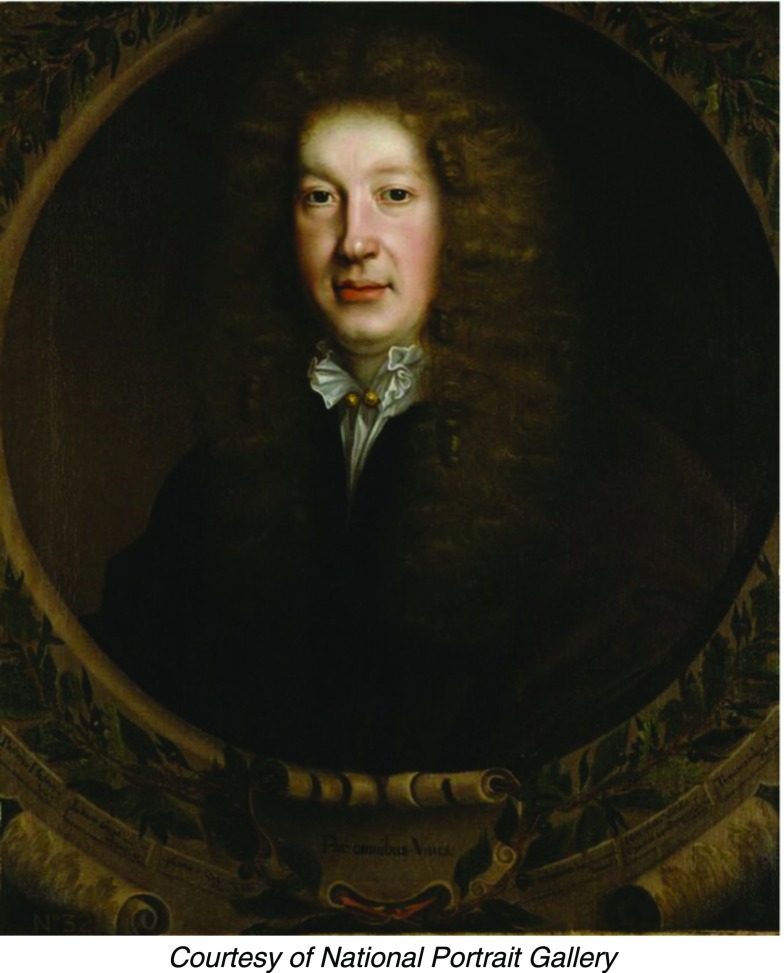



## Introduction

In 1667 the English poet, literary critic, translator, and playwright John Dryden published an historical poem entitled ‘annus mirabilis’ or ‘year of miracles’. Dryden was a key figure in Restoration England and wrote the poem whilst in Charlton in Wiltshire where he had taken his family to escape from the Great Plague in London, the last major outbreak of bubonic plague in England which killed 100,000 over an eighteen-month period. Indeed, the impact of the Great Plague was such that Charles II and the whole Royal Court left London firstly to Salisbury and then to Oxford where Parliament sat, leaving London in the hands of the Lord Mayor, Sir John Lawrence, and the aldermen of the city. Exodus for the poor was not as easy and it became increasingly difficult for the poor to leave as the plague ravaged the population.

Dryden’s poem was a celebration of the recovery of England from a series of events including not only the plague but also two wars and the Great Fire of London, the description of which formed the second half of the poem. It was considered that The Great Fire was in fact a miracle as the city was saved and Charles pledged to rebuild and improve those parts of the city that had been destroyed and indeed many of London’s most famous landmarks were built following the fire, such as Christopher Wren’s St Pauls cathedral and monument. The remodelled London was never again visited by bubonic plague which may in part have been due to the eradication of poor housing and creation of wider streets.

‘annus mirabilis’ was very well received by the King and in 1668 Dryden was made England’s first Poet Laureate, a title that is still in existence and which over the years has been held by such luminaries as Wordsworth, Tennyson, Masefield and more recently by John Betjeman and Ted Hughes.

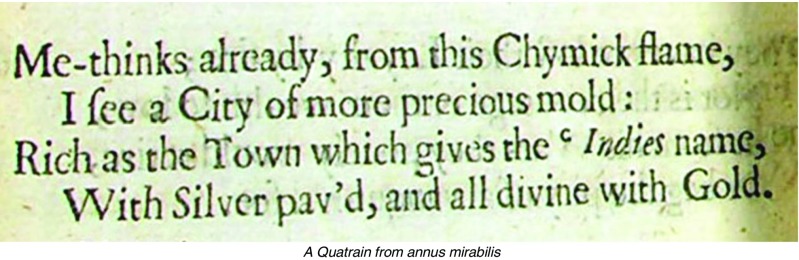



The poem ‘annus mirabilis’ was a display of optimism despite tragedy and an expression of the expectation of rebirth and better things to come. Dryden’s view was that God had saved London and England from certain destruction and therefore that things could certainly have been worse.

I have chosen this title in recognition of my year as President of the ISPN 2016–2017 and, like Dryden, I have hope and expectation that the situation of Global Neurosurgery for Children will improve in the years to come and the right of all children to be able to access safe and optimal surgical treatment for neurosurgical conditions will be achieved.

## Role of ISPN president

To be awarded the ISPN Poncho is a great honour and certainly from my own perspective it is the highest point of my career in paediatric neurosurgery. However, the term of presidency is brief and, therefore, to achieve any realistic goals during the period of tenure is only possible by continuity from one president to the next. I was extremely fortunate to have had as my predecessor Prof Graciela Zuccaro and as my successor Prof Graham Fieggen, both of whom share with me a vision of improved neurosurgical care for children on a global scale. It was through Prof Zuccaro that I became involved in the Global Initiative for Children’s Surgery (GICS) and during the course of her presidency, we had many conversations about the role of the ISPN in the context of global surgery and how we might improve membership in Low and Middle Income Countries (LMICs) according to the World Bank classification. We wanted to develop a strategy for the ISPN that could run through several presidential terms and, thus, it was essential that Graham Fieggen was also involved in these discussions.

In my role as ISPN President, I was very fortunate to be invited to contribute to scientific meetings, teaching courses for the ISPN and the ESPN and also to be a visiting professor in a number of places. From our very successful 44th annual meeting in Kobe hosted by Prof Mami Yamasaki, I travelled to Myanmar for an ISPN teaching course and this was followed by trips to India, Africa, North and South America, Russia, several European countries and New Zealand. During these travels I attempted to get some idea of the local circumstances and in particular the contrasts in man power and infrastructure in the countries and units that I visited. There are clearly many limitations to this as a process of information gathering but it did allow me to formulate some personal perspectives that I would like to share.

## Global health and global surgery

Global health has rightly become a very popular topic in recent years and is the health of populations in the global context; it has been defined as “the area of study, research and practice that places a priority on improving health and achieving equity in health for all people worldwide”.

It should be distinguished from public or international health and in its ambitions require not only multidisciplinary collaboration within medical disciplines but also other collaborations which allow the development of adequate infrastructure to facilitate medical care.

In 1978 the Alma Ata Declaration was adopted and the aim of ‘Health Care for All’ established. In 1980 Halfdan Mahler, then director-general of the World Health Organisation, first raised the issue of surgery as a part of health care for all but the current global surgery movement was born out the report of the Lancet Commission *Global Surgery* published in 2015 and the subsequent *Global Surgery 2030*. The commission demonstrated the huge inequity in the provision of surgical care, particularly in LMICs. Five billion people lack access to safe, affordable surgical and anaesthetic care when needed and an additional 143 million surgical procedures are needed to redress this deficit. Most importantly, it became recognised that economic growth is being impaired by the lack of adequate resources and an investment of $350 billion would result in an expected economic growth of $12 trillion. At a stroke, the importance of non-communicable disease has been recognised in economic terms and the economists and politicians are taking note. As Dr. Jim Yong Kim, President of the World Bank said in his opening address at the first meeting of the Lancet Commission on Global Surgery “Surgery is an indivisible, indispensable part of health care” and referred to surgery as “the neglected stepchild of Global Health”.

At the 68th World Health Assembly (WHA) meeting in 2015, Resolution 68.15 was formally adopted by the 194 member states of the WHO. The resolution was entitled “Strengthening Emergency and Essential Surgical Care and Anesthesia (EESA) as a Component of Universal Health Coverage” and is aimed at addressing the gaps in the provision of surgical and anaesthetic services. For the first time, it is being acknowledged that surgically treatable conditions are responsible for three times more deaths than malaria, tuberculosis and HIV/AIDS combined. The resolution acknowledges that surgical care should be an essential part of universal health coverage and urges governments to make plans for its implementation. The nine clauses of the resolution cover finance, data collection, manpower, education, provision of consumables, infection control and political involvement, by ensuring that Ministries of Health take a lead role in the promoting of EESA.

The ten most important needs for the provision of safe surgical and anaesthetic care have been further defined by the Lancet commission and the gap analysis performed has shown that LMICs are those with greatest need in terms of infrastructure, equipment, disposables and manpower. In addition the time taken to access surgical care was mapped and the deficiencies revealed, once again primarily in LMICs.

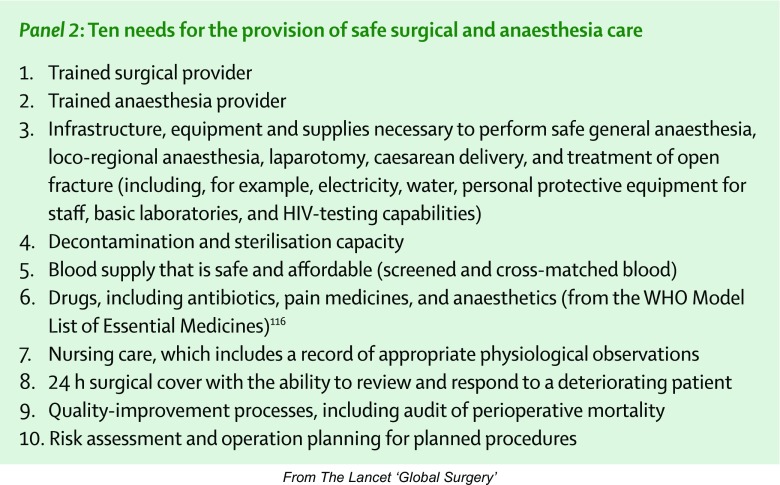



One of the concepts developed from this work has been the definition of *Bellwether* procedures. These are the procedures which can be used as indicators to gauge the degree of effectiveness of the provision of surgical care. For surgery as whole, they have been defined as caesarean section, laparotomy and treatment of open limb fractures with access to these interventions needed within 2 h. As yet Bellwether procedures for surgical specialities do not exist but if we want to gauge accessibility of the neurosurgical services that we supply to children then these procedures must be defined for our own speciality.

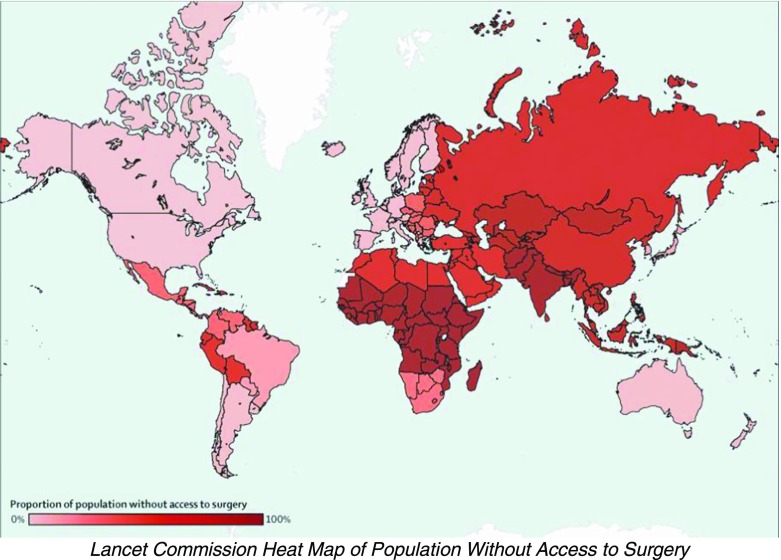

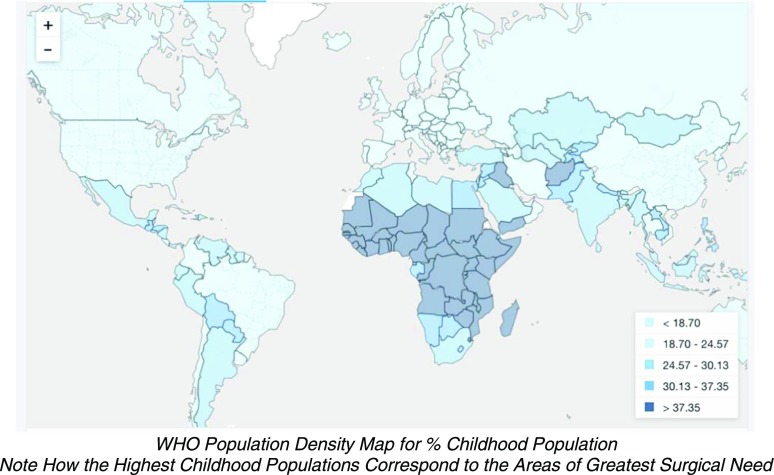





## The position of neurosurgery within global surgery

In the core packages for surgical and anaesthetic care described by the Lancet Commission the only neurosurgical procedure listed is “burr holes” for emergency care in the basic trauma package. No neurosurgical procedures of any sort feature elsewhere.

In 2015 “Essential Surgery—the World Bank Health Disease Priorities 3” was published and this recognises the importance of head trauma as both a major cause of death and disability with 10 million people suffering traumatic brain injury (TBI) globally every year. In addition, head injuries are commonest in LMICs and 90% of trauma deaths occur in LMICs. The guidelines for management have been, for the most part, drawn up in HICs and do not take into account the resource issues that affect LICs and that for many neuro trauma is often not an attractive surgical speciality. Burr holes again appear in the trauma procedures list whilst shunt for hydrocephalus appears in the congenital section, the only other neurosurgical procedure quoted.

WHO Guidelines for Essential Trauma Care published in 2004 was an attempt to create affordable and achievable basic standards of emergency trauma care and describes very well the situation present in many countries where, in the absence of neurosurgeons, TBI is managed by general practitioners and general surgeons. There are some excellent recommendations in this report but it acknowledges that the American Association of Neurological Surgeons (AANS) guidelines, considered to be the gold standard for head injury management, are not achievable in a low resource setting where even basic imaging and surgical facilities may not be present.

In their paper “Global Neurosurgery: The Unmet Need” Kee Park, Walt Johnson and Robert Dempsey have outlined the huge unmet need for neurosurgery. Whilst in HICs, the ratio of neurosurgeons is 1 per 80,000 population; data suggests that in LICs that ratio may be as low as 1 per 10 million population. This excellent paper is a “call to arms” for the neurosurgical community to become engaged in Global Surgery. It acknowledges the contribution of organisations such as Foundation for International Education in Neurosurgery (FIENS) and the World Federation of Neurosurgical Societies but also points out that neurosurgical representation in working groups on trauma has been minimal. We are doing well in the areas of neurosurgical education but that is not enough and the paper stresses the need for interdisciplinary working to develop necessary infrastructure and the need for academic research and publications on neurosurgery from LICs. Most importantly, the paper stresses that neurosurgeons need to become advocates for our speciality at a national and international level so that we can really address the unmet need. Neurosurgeons need to produce high quality recommendations appropriate for district level hospitals in LMICs and direct our educational efforts appropriately. Unfortunately, in the paper “Global Neurosurgery: The Unmet Need” there is no mention of children despite the fact that in many LMICs the under 18 population may be as high as 50% and that in LICs the childhood population is rapidly increasing. When it comes to the paediatric population, it follows that the deficiencies found in the provision of care for general neurosurgery are even more of a problem. As these countries are also those with the lowest density of neurosurgeons, there is little chance for true sub-specialisation in paediatric neurosurgery and care for children is therefore shared between paediatric general surgeons and adult trained neurosurgeons. Whilst in some situations, the care may be excellent. There is little doubt that without adequate training in the pathologies of childhood many children will receive poor care or no care at all.

## Global initiative for children’s surgery

In 2016 the ISPN was invited to be a stakeholder in the fledgling Global Initiative for Children’s Surgery (GICS). This initiative was created by a group of general paediatric surgeons who wished to explore the position of general surgery for children globally. GICS is therefore a consortium of providers, institutions, and allies from both the global north and the global south. It is an inclusive group which believes that children’s surgery includes all provision of surgical care to children, not just paediatric surgery and so GICS includes all specialties and subspecialties involved in children’s surgical care, such as plastic surgery, orthopaedics, anaesthesia, intensive care, radiology, pathology, laboratory medicine, paediatrics, nursing and physical and occupational therapy, as well as neurosurgery. By bringing together those that provide care with the policy makers and administrators, GICS aims to analyse the current state of surgical care in LMICs; develop global, regional, national and local priorities to improve the delivery of surgical care for children in LMICs; and identify and bring together resources to address those global, national, and regional priorities.

Two meetings were held in 2016, the first inaugural meeting at the Royal College of Surgeons, London and the second at the American College of Surgeons in Washington. As a result of platform presentations and break-out groups, an essential resources document was drafted which included some neurosurgical detail drafted by those neurosurgeons present. There was also a commitment on behalf of the ISPN to continue engaging with GICS and contribute to further meetings. I attended both of the GICS meetings and reported back to the executive board of the ISPN. I gave the opinion that whilst it was completely appropriate that we engage with adult neurosurgery in the Global Neurosurgery platform, GICS and our paediatric surgical colleagues share many of the same requirements in terms of resources and manpower as we do. It is for this reason that paediatric neurosurgery is, in most developed health care systems, carried out in the paediatric surgical setting within specialist paediatric hospitals rather than in adult neurosurgical wards and units.

During the two GICS meetings I met and discussed the provision of surgical care for neurosurgical conditions amongst general surgeons and learnt first-hand how poorly developed the speciality of neurosurgery is in some areas of the world and how much of the care for head injuries, acquired and congenital disorders of the nervous system is provided by general paediatric and orthopaedic surgeons. This reflected on my own experience when starting in Paediatric Neurosurgery in the United Kingdom in the 1980’s when our own sub-speciality was getting started and much congenital work was managed by our paediatric surgical colleagues. The links created through GICS enabled me to establish a dialogue with paediatric surgeons particularly in Sub Sharan Africa where the disparity between the need of the childhood population and provision of neurosurgical care is most pronounced.

## Yangon, Myanmar

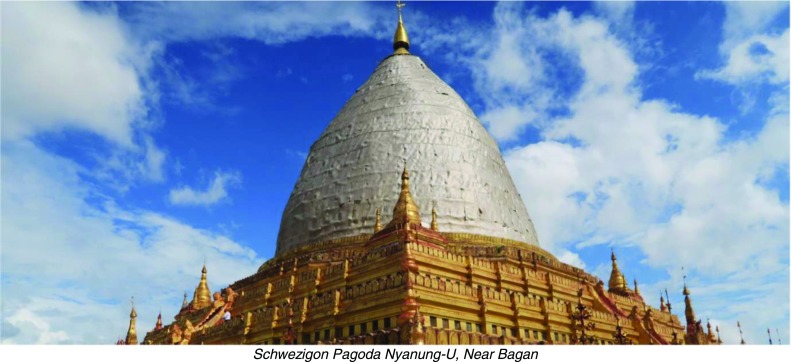



Immediately after the ISPN meeting in Kobe, we travelled to Yangon in Myanmar for the first ISPN teaching course of the presidential year and the first to be held in that country. In addition to an excellent ISPN faculty, we were joined by a number of adult colleagues with a history of collaboration with Myanmar. Notable amongst these was Dr. Jack Rock from Detroit who has worked with Prof Win on a number of occasions and is an active member of FIENS and their regional coordinator for Asia. FIENS has been active since 1969 and has placed volunteers in over 22 countries as well as working in partnership with other organisations such as the Neurosurgery and Development Foundation (NED) and the College of Surgeons of East, Central and Southern Africa (COSECSA).

Myanmar has been much in the News in recent months notably due to the plight of the Rohingya people, many of whom have fled to Bangladesh thereby creating a further humanitarian disaster, one accentuated by severe flooding. Myanmar is a country of some 54 million people of whom 10.8 million or 20% are children. According to WHO statistics, approximately 1% of the annual GDP of $1275.02 is spent on healthcare and the average life expectancy is 66.04 years. Thirty-six percent of the population is urban. There are 23 trained neurosurgeons in Myanmar but a further 30 in training and so the future looks optimistic. As yet, there are no subspecialist paediatric neurosurgeons although several of the trainees who attended our teaching course expressed a particular interest in paediatric neurosurgery. This stresses the need for education in paediatric neurosurgery pathology, clinical conditions and their management and finally the surgical procedures required to serve the 10.8 million children.

Prior to Military rule in 1962, the neurosurgical links were primarily with the UK but the Military government discouraged any outside contact and so there was a distinct change in medical education over the following 45 years. With the recent changes in the political picture in Myanmar, there has been active collaboration internationally and the ISPN teaching course marked a significant landmark in the development of neurosurgical care for children in the country. There are now active neurosurgical collaborations between Myanmar and the USA, India, Japan and Switzerland and I am pleased to report that the course was met with much enthusiasm and a further course is now planned for 2018. In addition, trainees from Myanmar are receiving training overseas and of particular note is the so called “South–South” links with India. South–South collaborations are where trainees receive fellowships in countries that have a similar socioeconomic structure to their own and therefore receive a more pertinent training.

### Take home message

After political isolation, effective collaborative partnerships have been created and the numbers of trained neurosurgeons is rapidly increasing. Paediatric neurosurgery does not yet exist as a sub-speciality but the ISPN teaching course is fulfilling an essential role both in stimulating interest in our sub-speciality as well as providing education. Collaboration with other countries in South Asia to create South–South partnerships has the prospect of developing training opportunities.

## Political role of the ISPN

This is perhaps an appropriate moment to discuss the political role of the ISPN. It is important to stress that the ISPN is an apolitical organisation and will never ally itself to any political party or regime. Our mission statement is clear that “The Mission of the ISPN is to improve the health and welfare of children requiring neurosurgical care throughout the world by scientific research and close international cooperation irrespective of class, colour, creed or economic condition”. The constitution and by-laws go on to state that we will strive to achieve this by:

1. Promoting and supporting effective social, clinical and scientific communication between paediatric neurosurgeons, basic scientists, political and governmental bodies throughout the world.
2. Developing and cementing relationships with other international organisations committed to the improvement in the health and welfare of the sick and underprivileged child.
3. Promoting and developing training schemes at a national and international level in paediatric neurosurgery supported by courses for developing countries and the provision of scholarships and exchange programs.
4. Supporting and promoting continued medical education within the society’s membership ensuring the maintenance of the highest levels of scientific and clinical knowledge.
5. Providing practical support to under-resourced colleagues working with underprivileged children in developing countries.

Both local and national politics can have a direct effect upon the health and well-being of children and particularly those with neurosurgical conditions. Whilst the ISPN is apolitical, it can and should contribute to political debate and lobbying either alone or by joining with other societies which share our interests. In particular the ISPN, through its executive board and membership, can comment on political matters affecting the neurosurgical care of children or on public health matters that have an impact on congenital or acquired conditions of the nervous system. Therefore, in matters determining the infrastructure within which we work, pertaining to child’s health and education and in matters of child protection, the ISPN should use its voice on behalf of children who otherwise will have no advocates. Children are not members of the electorate in any country and as such they cannot lobby for themselves and so they are often neglected by politicians. Examples of where the ISPN could have influence might be on the issue of folate supplements in the prevention of neural tube defects, Vitamin K injection at birth and head protection for sport and road use. We should help families in securing a safe and nurturing environment within which their children can develop and lobby so that payment at the point of delivery of surgical care does not result in severe financial hardship for families.

The history of neurosurgery is rich with examples of neurosurgeons who have made a major political contribution. Sir Victor Horsley supported movements as diverse as women’s suffrage, the Temperance League, anti-rabies legislation and the British Medical Association, all interspersed with highly significant surgical and physiological advances. He was also renowned for the kindness and understanding that he showed when looking after children. As his biographer remarked “Children understood and trusted him at once: he never chaffed or ‘talked down’ to them and though very gentle and pitiful, he was always bracing and straightforward with a young patient and he seemed able to really see from the child’s point of view”.

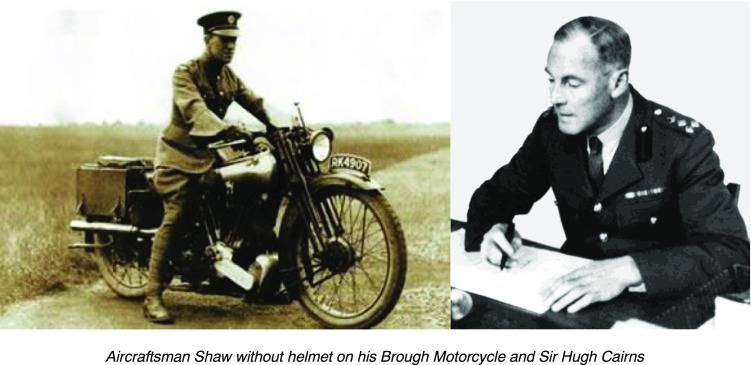



Sir Hugh Cairns did much to prevent injury to motorcyclists during World War II by lobbying for proper head protection following the death in 1935 of aircraftsman TE Shaw aka TE Lawrence or “Lawrence of Arabia”. Lawrence had been riding his motorcycle without a helmet and sustained fatal injuries when swerving to avoid two children. In a twenty-one-month period prior to World War II, there were 1884 fatalities amongst motorcyclists, two thirds due to head injury and the blackout restrictions of wartime led to a 20% increase in the death toll. Cairns called for the introduction of helmets and in 1941 the army, who had been losing two despatch riders a week, introduced compulsory helmets. More recently Hunt Batjer and Rich Ellenbogen have had an impact in the field of American Football through their work with the National Football League in the USA.

In the UK, Sam Galbraith became a prominent labour health spokesman whilst Balaji Sadasivan (Singapore), George Nga Ntafu (Malawi) and my friend Upendra Devkota (Nepal) have all achieved political eminence. In the United States, a paediatric neurosurgeon has even been a presidential candidate (Ben Carson) and is now Secretary of Housing and Urban Development in the Trump administration.

## Mumbai, India & the Ginde oration

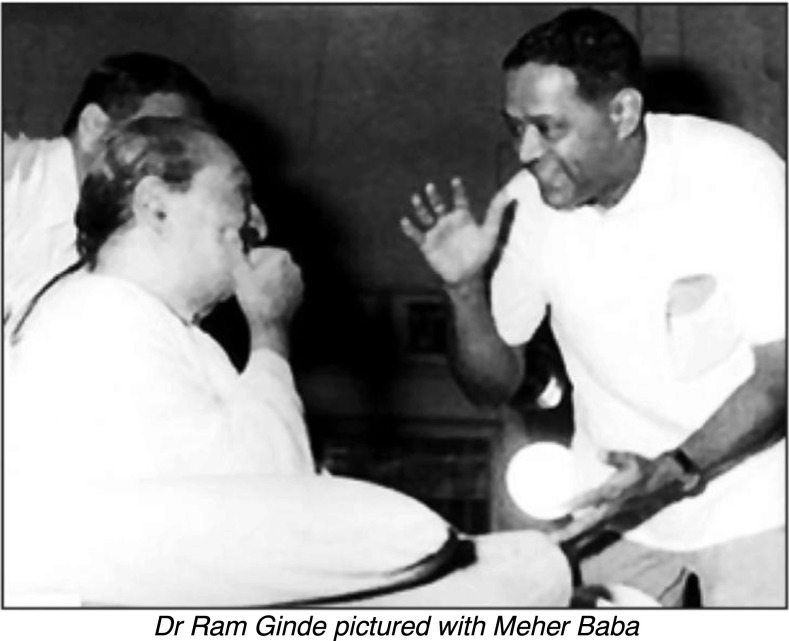



In March 2017, it was my very great honour and pleasure to deliver the Ginde Oration for 2017 during the 28th Annual Congress of the Indian Society for Pediatric Neurosurgery (IndSPN) held jointly with the 2nd meeting of the Asian-Australasian Society of Pediatric Neurosurgery (AASPN). Dr. Ram Ginde was a pioneer Indian Neurosurgeon who joined the staff at Bombay Hospital in 1953 and after whom the Oration was created by Prof Sanat Bagwati in 1991. Along with Jacob Chandy and B Ramamurthi, Ginde did much to further Neurosurgery in India and he was for many years editor of Neurology India and the representative of India at the WFNS for three terms. What is perhaps less well known is the relationship between Ram Ginde and Meher Baba.

Meher Baba was born in Pune to Irani Zoroastrian parents and devoted himself to mysticism from the age of 19 years and in 1923 opened his ashram in Meherabad where he opened a school, hospital and dispensary. From the age of 31 until his death aged 54 years, he maintained a code of silence, communicating by an alphabet board or hand gestures. He became popular in Western Culture from the 1940’s onwards and was a vocal opponent to the use of psychoactive drugs in the 1960’s. Meher Baba was involved in two road traffic accidents causing neck and neurological problems and he also suffered from trigeminal neuralgia, for which Ram Ginde was consulted and who subsequently became a lifelong friend and follower.

The subject of the Ginde Oration was within the field of Epilepsy Surgery and the contribution of the Neurosurgeon to the Multidisciplinary management team and this was given in the context of both anatomical dissections and a live surgery demonstration given by Dr. Sanjiv Bhatia from Miami and Prof Sarat Chandra of AIIMS, Dehli.

The history of paediatric neurosurgery in India is rich with the names of ISPN past presidents such as Sanat Bhagwati and Chandrashekhar Deopujari. In his presidential address during the 43rd ISPN meeting in Izmir in 2015, Prof Deopujari spoke about the history of the ancient civilisations concentrating on India, its problems associated with delivery of healthcare and the vision for the future which is undoubtedly a bright one. The IndSPN has a membership of over 200 on a background of some 1800 neurosurgeons in India, catering for a population of 1.4 billion inhabitants. Life expectancy amongst the Indian population is 68.8 years whilst 1.4% of the annual GDP per capita of $6,09.60 is spent on healthcare. Forty-one percent or 518 million of the population are under 18 years and delivery of care is complicated by 32% of the population being urban and that there are over 20 official languages within India. The Lancet Commission commenting on delivery of surgical care for abdominal emergencies in India stated that the mortality risk was 16 times higher when living 100 km or more from a well-resourced hospital, which demonstrates the challenges of delivery of care that such a huge country presents. This must be even more so for neurosurgical care where a workforce of 1800 is expected to deliver care to a population of 1.4 billion with 21 official languages.

From a political standpoint, India is the world’s largest democracy and the current Bharatiya Janata Party (BJP) under Prime Minister Narenda Modi has pledged to increase the current health spending to 2.5% GDP. Neurosurgical training in India has very much followed the lines of training in the UK but there are few paediatric neurosurgical fellowships and few paediatric neurosurgical departments, again a reflection of the paucity of trained neurosurgeons generally. In the past, medically and neurosurgically trained Indian nationals played an important part in delivery of care in the UK National Health Service, joining the junior surgical staff in the delivery of clinical care whilst at the same time expanding their surgical knowledge and expertise. Indian graduates would then return to India to resume their clinical positions. This tradition was changed by the UK joining the European Community as positions had to be preferentially offered to European graduates before graduates from the former commonwealth. This led to considerable dissatisfaction from both the neurosurgical units in the UK and the Indian graduates who were effectively blocked from entering the UK to gain further training. Following the referendum in the UK and the decision to leave Europe, discussions have already begun about how these traditional links can be re-established in neurosurgery for the benefit of both our populations.

### Take home message

The numbers of neurosurgeons in India remain inadequate for the size of population and fellowships in paediatric neurosurgery are needed to ensure adequate training in the speciality. The IndSPN is very active and encourages ISPN teaching courses through their own cycle of education. Excellent teaching programmes within neurosurgical units and centres of excellence in India offer a suitable resource for South–South collaboration within Asia.

## Kathmandu, Nepal

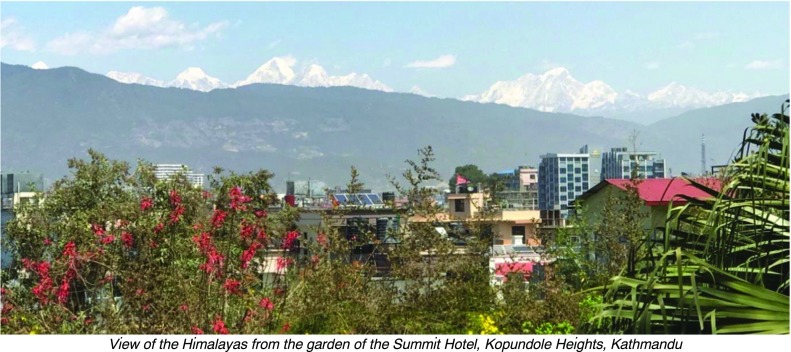



I first visited Nepal in 1999 to attend the South Asian Neurosurgical Congress organised by Upendra Devkota. Since childhood, I had wished to visit the mountain kingdom which has always had a special place in the heart of the British people. My great friend and housemate from University, Martin Entwistle, had joined the Royal Army Medical Corps after graduation and I had lunched in the Gurkha Mess in Aldershot on the day immediately before my wedding. The tales of bravery concerning these quiet people are legendary and acknowledged in the extraordinary number of honours awarded to Gurkha soldiers, including no less than 26 Victoria Crosses conferred on Gurhka officers and men. Since that first visit, I have been trekking in Nepal on a number of occasions and have grown to love the landscape and its people. On May 29th 2008, after many years of political upheaval, the Federal Republic of Nepal was declared and since then a new constitution has been agreed.

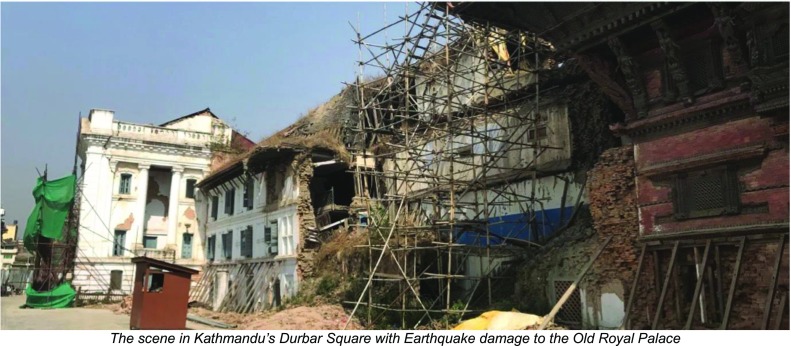



Nepal was hit by a series of earthquakes and aftershocks in April 2015 which killed nearly 9000 people and injured approximately 22,000. The natural catastrophe was made worse by 3.5 million being rendered homeless due to the collapse of buildings and the destruction of the fragile communications links within the country. Kathmandu was badly affected with several World Heritage sites being severely damaged.

For this visit, I was accompanied by my youngest daughter (aged 20 years) and the purpose of the trip was to visit some schools being rebuilt by the “In Your Hands” charity. We also travelled out to the worst hit rural areas in Bamti Bhandar, a fourteen-hour drive from Kathmandu, where we visited a school and first aid post. Nepal has a well-developed infrastructure of health posts and primary health centres managed by the Village Development Committees (VDC). However, the earthquake, aftershocks and subsequent flooding had rendered many roads impassable and even though our visit was two years after the event, the evidence of the damage was everywhere and the further away from Kathmandu that we travelled, the less visible impact of the $4 billion in aid money was evident. The bureaucratic process for restitution appeared complex and building supplies and experience both expensive and in short supply.

The Federal Republic of Nepal has a population of 29.3 million, of whom 12.3m or 42% are under 18 yrs. Life Expectancy in Nepal is 70.7 years whilst the GDP per capita $729.53, of which 2.3% is spent on healthcare. There are a total of 50 trained neurosurgeons and 8 residents in training and it therefore seems clear that the numbers of trained neurosurgeons and also those in training are inadequate. Twenty percent of the population is urban and 7% of the population live in the Himalayas whereas the concentration of specialist surgical help is necessarily located in the towns. This means that the provision of neurosurgical care is woefully inadequate and many patients have to travel considerable distances in hostile geographical circumstances and are then frequently lost to follow-up.

At present provision of paediatric neurosurgical care is confined to trauma, myelomeningocele and hydrocephalus with few resources for the adequate treatment of other conditions.

The new federal government of Nepal is committed to resolution 68.15 and has a Joint Country Cooperation Strategy with the WHO. However, at present the words “Too Many, Too Far, Too Poor, Too Late, Too Few to Help, Too Little Done” apply to Nepal and it is only with considerable additional investment in healthcare infrastructure that the objectives of 68.15 can be achieved. One solution may be investment in the natural resources of Nepal, particularly hydroelectric power, whilst to deal with communications and transport problems, the use of digital technology, particularly by the use of telemedicine, may ensure a more equitable distribution of healthcare.

### Take home message

One of the poorest countries in the world with access to surgical care of any sort is hampered by geography as well as lack of infrastructure and personnel. The new Federal Government is actively looking for development partners and it is this that potentially gives the greatest feeling of optimism for the country.

## Los Angeles, USA

When I was first appointed as a Consultant Neurosurgeon to the National Hospital, Queen Square and to Great Ormond Street Hospital for Sick children, I was given financial support and two scholarships through the Royal College of Surgeons England to visit North America and learn about Epilepsy surgery. For six months in 1991, I was a visiting Professor at University of California in Los Angeles (UCLA) and also visited Miami, Dallas, Seattle and Montreal, although the longest time was spent in Los Angeles where we stayed with our young family. It was an invaluable experience both professionally and personally. The Adult Epilepsy service was run by Prof J “Pete” Engel whilst the Paediatric service was run by Don Shields and both of these academic giants were very much in favour of evolving the surgical side of epilepsy treatment. The paediatric neurosurgeon at the time was Warwick Peacock of dorsal rhizotomy fame. In Miami I was very warmly received by Michael Duchowny, Prasanna Jayakar and Trevor Resnick and it began a long and very fruitful relationship between our academic departments. The final part of my six months sabbatical was spent in Montreal with Andre Olivier and Fred Andermann whose Professional expertise were second to none and whose personal kindness and consideration faultless.

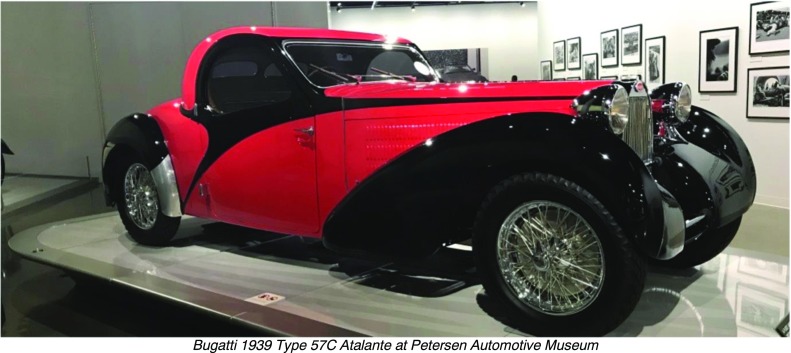



Being no stranger to Los Angeles, it was a great pleasure to receive and accept an invitation to speak in the International section of the AANS meeting hosted by Dr. Frederick Boop. It also allowed me the opportunity to visit the incredible Petersen Automotive Museum, where the special display was of an amazing collection of Bugatti cars. An extraordinary visit further enhanced by a visit behind the scenes to the collection of cars not normally on open display.

The United States has a population of 323.1m of which 73.6m or 23% are under 18 years. Life expectancy 79 years, GDP per capita $57,466.79, 17.1% GDP spent on healthcare or the equivalent of $10,000 per capita per year but as pointed out in the document “To Err is Human” high spending on healthcare does not necessarily result in improved care and many are unable to access affordable surgical care. Birth rate of the population is declining with a resultant increase in elderly retired population, no longer actively contributing to the taxation pool.

This population of 323.1m is served by 4200 neurosurgeons of which 220 board certified in paediatrics, a similar number to the neurosurgeons who are members of the IndSPN serving a population of 1.4 billion. There is a very well integrated infrastructure although universal healthcare in not established and appears to be a political bagatelle. The current administration has recently created barriers to free travel to the USA on the grounds of homeland security and this meant that a number of ISPN members who had papers accepted could not attend and others chose not to attend.

Rick Boop is a world recognised Paediatric Neurosurgeon, active ISPN member and an authority on paediatric oncology and as President of the AANS he made the theme of the meeting “Global Neurosurgery”. There was an excellent and inspiring opening session on the Global Neurosurgery theme and from the presentations made it was clear that there are a number of Global surgical Initiatives in Neurosurgery based in the US and that they have been doing excellent work, in some cases for several decades and certainly prior to the report of the Lancet Commission. However, a great number of agencies are involved in this work with little or no ability to cross reference or compare notes on successful or more importantly unsuccessful projects.

In the international section, I was able to put forward the hypothesis that for Global Neurosurgery to reach the childhood population effectively the ISPN had a role in collaboration with other agencies. After the session a number of us met to discuss what steps could be taken to advance Global Neurosurgical care with specific reference to children. During these discussions, it became apparent that little was truly known about the global neurosurgical workforce for children, the level of expertise and facilities available and the plans in place to correct any deficiencies. In conjunction with Prof James Johnston from the University of Alabama at Birmingham and Dr. Michael Dewan from Vanderbilt University in Nashville, we decided to take it upon ourselves to develop two initiatives; first, to carry out a survey in an attempt to get an idea of the current status of paediatric neurosurgical care and its delivery and second, to see if we could develop a platform for establishing collaborative partnerships.

### International survey

This was created by Michael Dewan and collated using RedCap and distributed to members of the ISPN, GICS, IndSPN, AASPN and other organisations during July and August of 2017. A full description of the outcome of this survey will be given by Michael Dewan in the following presentation and is to be submitted for publication but in brief we received 512 responses from 78 countries. Of the 512 responses 405 were from neurosurgeons of whom 338 described themselves as having a major commitment to paediatric neurosurgery. There were responses from 107 non neurosurgeons and the questionnaire explored issues of training, manpower and resources.

Although the survey was not exhaustive and clearly has limitations, it has given a vignette of the huge disparity of manpower, facilities and skills and does not paint a good picture for the future. From this survey, a number of key issues have been raised.

### Matching website

In the survey that we carried out, we asked respondents if they would be interested in international collaboration and over 80% said that they would. This has led to the creation of InterSurgeon of which I will speak later.

### Take home message

The United States is resource and personnel rich but despite this there are those that are disadvantaged and universal healthcare is not present to all strata of society. There is an excellent record of humanitarian work being carried out by paediatric neurosurgeons and their adult colleagues but there is a need for collaboration and spread of experience and information.

## Pietermaritzburg, Republic of South Africa

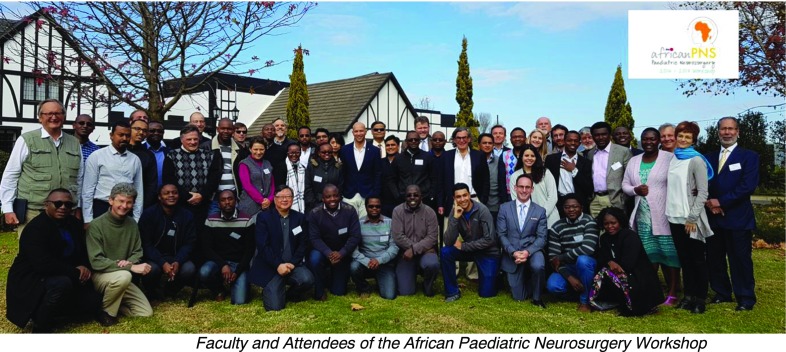



In June 2017 I was invited by the ISPN President Elect Prof Graham Fieggen, to take part in the 2nd African Paediatric Neurosurgical Workshop. This is an excellent course run for the benefit of all African countries and is strongly supported by both the ISPN and the ESPN. Themes for the meeting included epilepsy and neuromonitoring and the invited faculty brought with them unparalleled experience. It was a particular pleasure for me to take part in the course as my paternal grandfather was born and died in Pietermaritzburg, after my great grandfather settled there in the latter part of the nineteenth century. I was, therefore, able to visit family graves and houses once occupied by my father’s family, as well as the regimental headquarters of the Natal Carabineers, a regiment to which my great grandfather had been a bandsman.

Pietermaritzburg has another peculiar place in history as it was on the platform at Pietermaritzburg station in June 1893 that Mahatma Ghandi, a young lawyer, was ejected from a first class carriage, an incident that started his political career.

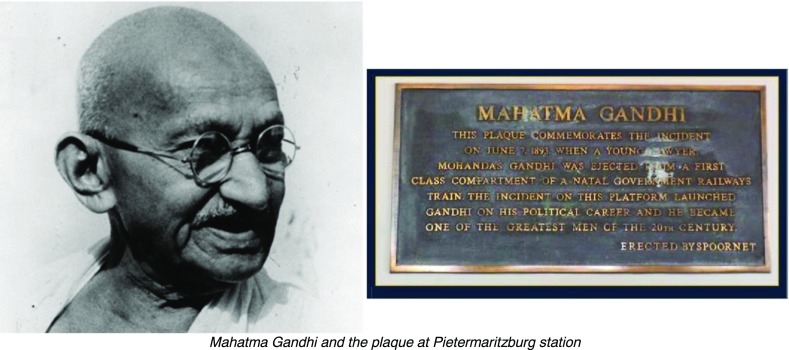



South Africa (RSA) has taken a prominent place in Neurosurgical education in the African Continent particularly the University of Cape Town and it has had a previous ISPN President Jonathan Peter as well as hosted a remarkably successful 36th annual ISPN meeting in Cape Town in 2008. RSA has a population of 56.9 million of whom 18.5 million or 34% are under 18 years and of whom 64% live in poverty. Life Expectancy is only 50.3 years and of the GDP per capita of $5273.60, 8.8% is spent on healthcare. There are approximately 180 neurosurgeons of whom only 4 are in full time paediatric neurosurgical practice. Despite socioeconomic hardships and political turmoil, RSA is playing a principal role in education in Sub Saharan Africa (SSA) where the issues are immense. The population of SSA already exceeds 1000 million and of this 50% are under 18 years. The birth rate is continuing to increase and it is anticipated that by 2050 one in three of the world’s children will be in SSA. To serve this huge childhood population there are less than 15 neurosurgeons trained specifically in paediatrics. The World Federation of Neurosurgical Societies created a training centre in Rabat which has trained a number of surgeons in SSA and this work has led to training centres and programmes developing in many other countries as well. The College of Surgeons of East, Central and Southern Africa (COSECSA) is taking a lead in running courses on basic surgical skills, including neurosurgery and in neurotrauma.

### Take home message

Africa and particularly SSA has a crisis of delivery of surgical care which can only be resolved by developing innovative solutions to creating infrastructure and by workforce optimisation in neurosurgery.

## Buenos Aires, Argentina

In July 2017, Prof Roberto Jaimovich invited me to be the first visiting professor since he took over from Prof Graciela Zuccaro as head of Neurosurgery at Hospital de Pediatria Garrahan. Despite its recent economic troubles, Argentina has a long-standing commitment to paediatric neurosurgery beginning with Raul Carrea, a founder member of the ISPN and President from 1978 to 1979. Argentina has a population of 44.4 million of whom 13.3 million or 23% are under 18 years. Life expectancy 77.8 years, GDP per capita is $12,449.22 of which 8% is spent on healthcare. The unusual thing about Argentina is that there are approximately 1000 trained neurosurgeons and of these 70 are full time paediatric neurosurgeons.

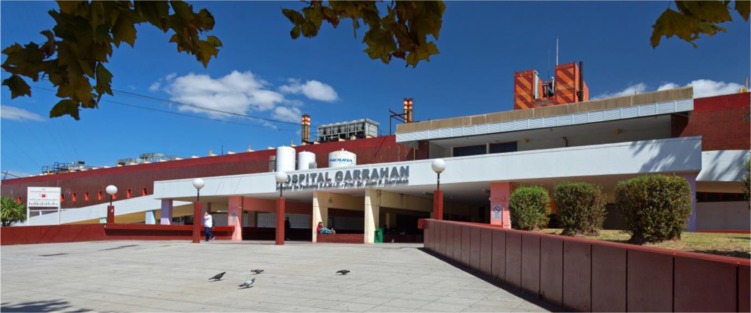



I have visited Buenos Aires and Hospital Garrahan on a number of occasions and through the epilepsy surgery team of Dr. Hugo Pomata had the pleasure of receiving Marcello Bartolucci in London for a period of six months, since which time he has made great progress in epilepsy surgery in Buenos Aires.

Garrahan has an extremely active training programme and there are two features that make it unusual. The first is that the residency is in paediatric neurosurgery alone with only a few months spent with the adult neurosurgical programme, which means that once trained, the residents are committed to a future in paediatric neurosurgery alone. Secondly, the programme accepts two trainees from Colombia on a regular basis and so is creating strong links within South America and improving the standard of neurosurgical care for children outside Argentina. As well as giving lectures and attending the operating sessions, I was able to spend time with the residents who were very well motivated and had clear ideas about possible improvements to the training programme which I was able to feed back to Prof Jaimovich. The unit at Garrahan is young and enthusiastic and has a significant role to play in education in South America and in collaborations through the Federacion Latinoamericana de Sociedades de Neurochirugia (FLANC) and PediFLANC its paediatric arm.

### Take home message

Paediatric neurosurgery in Garrahan is taught in isolation from adult neurosurgery and the teaching programme welcomes trainees from outside Argentina.

## Santarem, Brasil

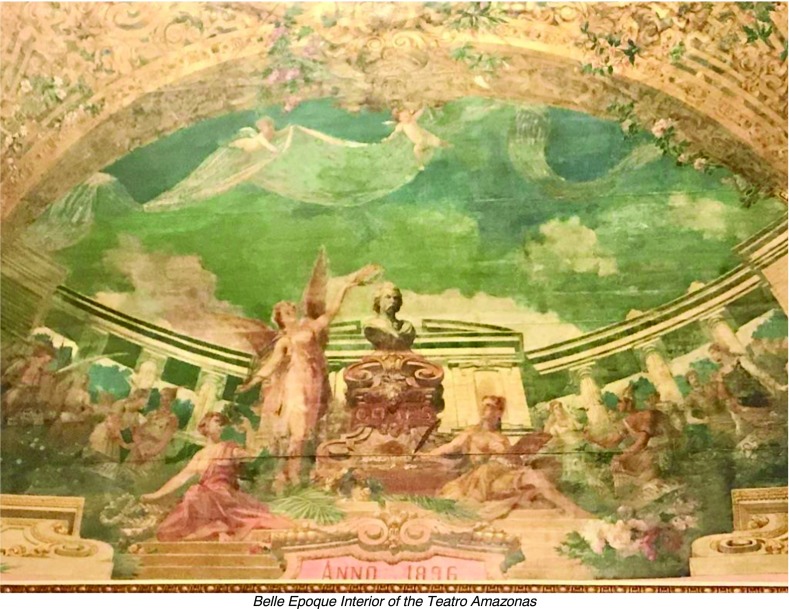



In August, an ISPN teaching course was organised in Santarem, a city in the Amazon region of Brazil. It was a wonderful opportunity to visit an area of Brazil to which we had not previously travelled and to fulfil an ambition to visit the opera house in Manaus. One of my favourite movies is Fitzcarraldo, the extraordinary film by Werner Herzog which tells of Brian Sweeney Fitzgerald (Klaus Kinsky) who wishes to build an opera house in Iquitos, Peru. The film begins with Fitzgerald and Molly (Claudia Cardinale) attending the opera in Manaus to hear Enrico Caruso sing. The opera house was built in the Belle Epoque at the end of the nineteenth century at the height of the rubber boom and many of the materials to build it were brought from Europe. It was said that the intention was to attract Caruso to sing at the opening of the Opera House in January 1897 but whether he did actually sing there now seems to be a matter for debate. However, we were able to attend a concert in this magnificent pink building and another dream was realised.

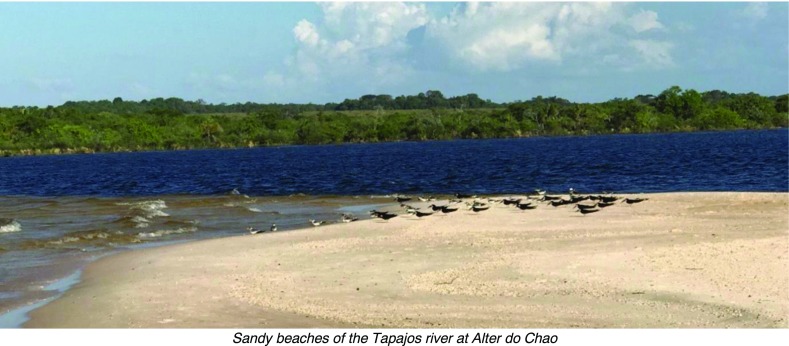



From Manaus we travelled down to Amazon by boat to Santarem where the course was being held, hosted by Dr. Erik Jennings Simoes. The venue was the splendid auditorium created by Erik’s brother near the town of Alter do Chao on the Tapajos river. The scenery was fantastic as too was the hospitality and the quality of the course outstanding. Dr. Simoes has a long history of working with the indigenous tribes in the interior of the Amazon region and has for many years been working with the Zoe tribe who he visits on a regular basis to provide medical care. We had the unique experience of meeting a Zoe tribesman during the conference as one of the tribes’ foremost hunters fell from a tree and had to be brought to Santarem to be checked out. The tribe are identifiable by their long lip plugs and only came in contact with the outside world as recently as 1982.

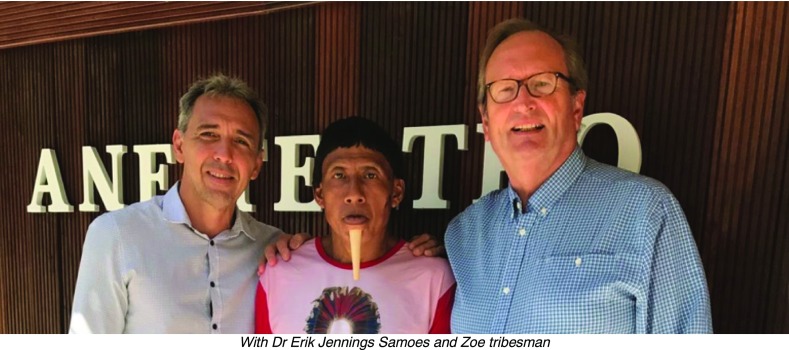



Brazil has a population of 209.5 million of which 62.85 or 30% are under the age of 18 years. Life expectancy is 73.8 years and the GDP per capita is $8649.95, of which 8.3% is spent on healthcare. Brazil has a well-developed infrastructure with 2875 neurosurgeons of whom 60 are primarily paediatric neurosurgeons. Over the last two decades there has been significant growth in the Brazilian economy, which has brought prosperity but in recent years this growth has slowed and the country has become embroiled in a number of political and financial scandals, centred around bribery and corruption. Although Brazil has enjoyed economic success and has a well-developed infrastructure there is a concentration of population and resources to the major conurbations which means that there are many areas which are geographically remote and where neurosurgical services for children are poor. This was demonstrated by our hosts in Santarem who cover a huge geographical area with many of the remote areas inaccessible except by boat and therefore treatment delays inevitable.

### Take home message

Despite its recent economic success many areas of Brazil are poorly served with paediatric neurosurgery. There needs to be some incentive to working in more isolated and remote areas and develop infrastructure to support doctors in these regions. This geographical inaccessibility has parallels with Nepal although economically they are very dissimilar. Nonetheless, like Nepal, one solution may come through training programmes and the use of digital technology both for delivery of clinical care and for education.

## Denver, Colorado

The end of my presidential year is marked by the 45th Annual ISPN meeting in Denver at which this speech is delivered. Denver is in fact another city with which I have family ties, although this time on my maternal side. My maternal great great grandfather was a mining expert from Cornwall in England who was invited to go out to Denver to improve the yield from the extraction of gold from ore, as after the initial seams of gold had been worked, the deeper veins of gold were mixed with copper and iron pyrites, making extraction much more costly. After an initial visit to assess the situation Richard Pearce was offered the job of setting up new furnaces firstly at Empire and then Black Hawk. He then lived with his family for many years in the Denver area, becoming British Vice Consul and receiving an honorary PhD from Colombia College New York. My Grandfather was born in Denver but as the mining business declined in Denver, his family returned to Cornwall when he was 7 and so apart from a brief period at the Massachusetts Institute of Technology following World War I in 1919–1920, he spent the rest of his life in England.

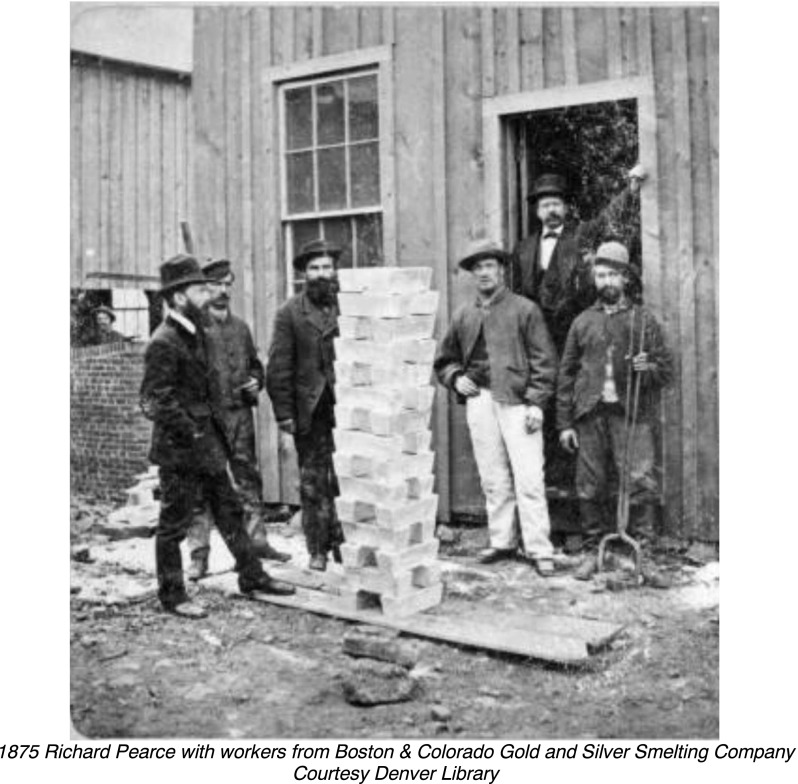



During 2017 and following the AANS meeting, Jim Johnston and I worked on the idea for a matching site for international collaboration. Whilst listening to the presentations at the AANS meeting in Los Angeles earlier in the year, I was struck by the fact that although there was excellent work being done in many parts of the world it was uncoordinated. A study carried out by the International Education Subcommittee of the AANS/CNS joint paediatric section and published in *J Neurosurgery Pediatrics* showed that a 29 separate agencies were involved in different forms of collaboration. Of 116 respondents 61% had carried out or taught neurosurgery in a developing country, with 49% travelling annually to do so. Seventy-seven percent expressed the wish to work elsewhere but 43% stated that inability to identify a collaborative partner prevented them from doing so. They concluded “Creation and curation of an online database of ongoing projects to facilitate coordination and involvement may be beneficial”. However, a simple mapping process has considerable difficulties associated with it and Dr. Marilyn Butler of Global Pediatric Surgery Network was able to share with us the difficulties she has encountered over the last ten years in establishing a mapping project for general paediatric surgery.

We therefore decided to develop in independent web-based platform to match centres offering services to those in need of assistance. We went through a branding process for the project and then developed the website along the lines of a dating site. With support from the ISPN, the University of Alabama at Birmingham and private donations the InterSurgeon project has been born and is continuing to be developed, in the hope and expectation that more partnerships can be created within paediatric neurosurgery and thereby children’s lives improved. InterSurgeon will be free to all and our intention is to register as a charity in the UK.

### Models of partnership & fellowship

In the session following this presidential address, we will hear from several people who have extensive experience of long-term international collaborative partnerships and each has evolved a different model appropriate for their circumstances. Prof Michel Zerah has worked for many years in Vietnam, teaching paediatric neurosurgery to adult neurosurgeons. Prof Ezio di Rocco has been teaching third ventriculostomy to general surgeons whilst Jim Johnston has applied modern technology in the form of a tablet to provide remote surgical supervision, advice and teaching in Vietnam.

Dr. Maya Bhattachan from Kathmandu works alone in her institution and wishes to have a brief period of intensive exposure to surgical procedures in a South–South partnership in order to observe specific techniques not easily understood from the textbooks. She will describe how her neurosurgical practice consists primarily of traumatic brain injury as a result of poor levels of child safety and that the majority of tumours present very late and how there is very poor follow-up.

Patrick Kamalo from Malawi and Gyang Bot from Nigeria both have experience of International fellowships but explain the difficulties of applying what they have learnt to their local, resource-poor situation.

I hope that these presentations and the discussions that follow will stimulate us all to consider what part we can play in Global Neurosurgery for Children. The key issues are to expand the neurosurgical workforce, whilst at the same time optimising the workforce already in place; collaborate with others to develop better infrastructures and create training schemes that deliver care as close to the point of need as possible.

## Travelogue summary

*One’s destination is never a place, but a new way of seeing things—Henry Miller*

I have not included in this address every country or place that I visited during my presidential year, despite the fact that the process of travel and every destination visited has given me an idea or firmed up my resolve on ideas previously formulated. The ISPN already contributes to education in our speciality by organising teaching courses in many parts of the world and these have now extended to include nurses. The ISPN Guide is an electronic text accessible without charge through the ISPN website. We recognise that the ISPN annual meeting is a platform for exchange of information and meeting others who may be working in similar environments. For this reason, we give financial support to attend the annual scientific meetings through scholarships and reduced fees for LMIC attendees and offer candidate membership of the society with access to Child’s Nervous system free of charge. We offer finance for visiting fellowships and observerships both for individuals and multidisciplinary teams. Finally, there are reduced membership fees offered for applicants from LMICs. The ISPN has also provided funding for the InterSurgeon project which offers the potential to create collaborative partnerships.

## The question now remains ‘What else can the ISPN do?’

There is no doubt that the ISPN does a lot to further Global Neurosurgery for children but we can and should do more. I believe that we need to engage further with other organisations involved in Global Surgery and in particular continue our association with GICS. This will offer to us the opportunity of bringing paediatric neurosurgery education to paediatric surgeons who represent the greatest opportunity for improving delivery of neurosurgical care in many parts of the world for conditions such as TBI, hydrocephalus and myelomeningocele. We must overcome the professional isolation that many neurosurgeons seem to think is an obligatory part of our speciality and recognise that if we increase the amount of task sharing or workforce optimisation that we engage in, it is likely that we will introduce our speciality to a wider audience and in so doing strengthen rather than weaken our speciality. This clearly needs to be in the context of training more neurosurgeons appropriately for their local situation but unless we take advantage of the skilled workforce present in the form of general paediatric surgeons, many lives will be lost. In some countries, it will take several generations before the numbers of neurosurgeons will be sufficient to provide universal health coverage.

GICS also gives us the opportunity to get paediatric neurosurgery recognised as part of the National Surgical Plans of LICs bringing to the attention of the politicians what our speciality can offer and the potential savings of life and money that can ensue from neurosurgery for children being a part of a well-planned national surgical strategy. We must be involved in the implementation of WHA resolution of 68.15.

We have the opportunity through the WFNS to improve instrumentation in many parts of the world and we should advise on equipment suitable for paediatric use. We need to liaise more closely with the WFNS over the paediatric content of their educational courses and indeed integrate them into the work of the ISPN Educational Committee. The WFNS is also recognised globally as our international representative professional body and we should seek a means to be more fairly represented by them as currently the ISPN is not itself a member of the WFNS. Through the WFNS, we should lobby other organisations such as the WHO on matters relating to the neurosurgical wellbeing of children such as dietary supplementation with folate, Vitamin K injection at birth and head protection and restraints during sport and road travel.

Finally, InterSurgeon offers a platform for international collaboration and as the work on this project progresses every ISPN member who is interested in the matter of Global Neurosurgery for Children should enrol and create offers or requests so that International Partnerships can be created and the plight of children with neurosurgically treatable conditions improved.

## 2016–2017 annus mirabilis

In conclusion I believe that the ISPN has a unique role in both education and in political lobbying and that by working in concert with others we have the opportunity of having significant and long lasting impact on the prevention and surgical management of lesions of the central nervous system in children and in so doing, improve the length and quality of their lives.

William Harkness MB ChB FRCS

ISPN President

Cornwall, England

With grateful thanks to and warm appreciation of

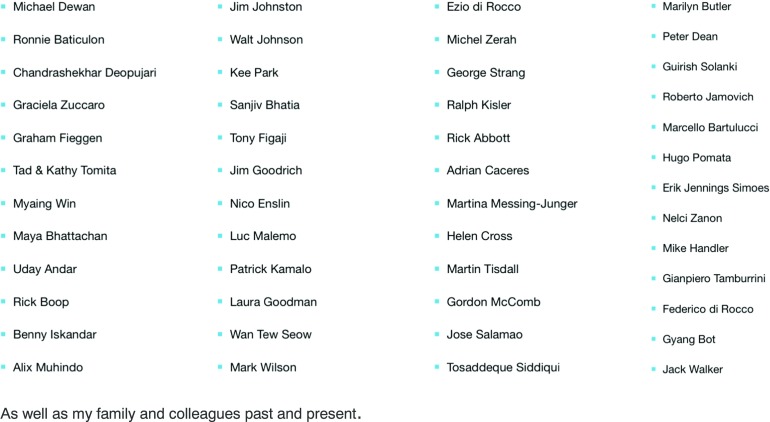



### In memoriam

In my text and in the list of thanks above, I have acknowledged my friend and colleague Dr. Sanjiv Bhatia. In May of 2018, Sanjiv died under tragic circumstances and the whole of the paediatric neurosurgical community is still reeling from this. Sanjiv was a masterful surgeon, a great thinker, an excellent educator but most of all a wonderfully warm and caring individual who loved his patients and doted on his family. He had worked in Haiti with the relief programme of his colleague John Ragheb and was one of the great supporters of Global Neurosurgery for Children and joined us for the discussions at the AANS meeting in Los Angeles where our survey and InterSurgeon were born. We have lost a great friend and wonderful colleague.

### Addendum


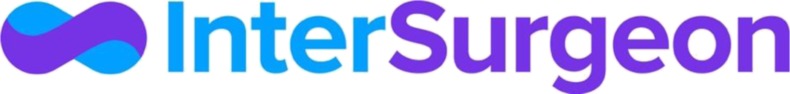


Since the ISPN meeting in Denver the InterSurgeon website has been through testing and it was launched on March 15th 2018. In the first three months, we have had over 160 members from 52 countries join the website. We have been asked by the WFNS to expand the membership of InterSurgeon to adult neurosurgery and are actively seeking funds to do this. We also hope to expand into Paediatric Surgery and then Urology later in 2018.

Although now retired from clinical practice, I am honoured to be appointed as the ISPN Ambassador for Global Neurosurgery and have been nominated as a member of the WFNS-WHO liaison committee and recently attended the 71st World Health Assembly meeting in Geneva. I attended the satellite symposium on Global Surgical, Obstetric and Anaesthetic care, a technical meeting hosted by the WHO Essential and Emergency Surgical Care Programme. In addition, I have continued to travel visiting Peru in December 2017 to carry out a review of the paediatric neurosurgical service at Instituto Nacional de Salud del Nino, Brena, Lima. I took part in the first ISPN teaching course to be held in Bangladesh in January 2018 and the third GICS meeting which was held in Vellore, India in January 2018, during which I gave a very brief neurosurgical skills presentation to general paediatric surgeons with a hands-on demonstration using a Hudson brace and Gigli saw.

## Sources

The material for this presidential address was derived from a large number of medical and non- medical sources. These included the WHO and World Bank website data for population and healthcare statistics. Essential reading on the subject of Global Surgery include

1. WHO Programme on Neurological Diseases and Neuroscience, World Health Organization. (2004). Atlas : of a collaborative study of the World Health Organization and the World Federation of Neurology. Geneva : World Health Organization http://www.who.int/iris/handle/10665/43075
2. Global Surgery 2030: evidence and solutions for achieving health, welfare, and economic development Meara, John G et al. Lancet , Volume 386 , Issue 9993 , 569–624
3. Disease control priorities 3rd edition. Volume 1: Essential surgery. Edited by Haile Debas, Charles Mock, Atul Gawande, Dean T Jamison, Margaret Kruk, and Peter Donkor, with a foreword by Paul Farmer
4. Guidelines for essential trauma care. Mock C, Lormand JD, Goosen J, Joshipura M, Peden M. Geneva, World Health Organization 2004
5. Essential surgery: key messages from disease control priorities, 3 Charles N Mock, Peter Donkor, Atul Gawande, Dean T Jamison, Margaret E Kruk, Haile T Debas, for the DCP3 Essential Surgery Author Group
6. Presidential address 2015 . C Deopujari. Childs Nerv Syst (2016) 32:1761–1767
7. Global surgery: defining an emerging global health field. AJ Dare, CE Grimes, R Gillies, SLM Greenberg, L Hagander, JG Meara, AJM Leather Lancet 2014; 384: 2245–47
8. Effect of geopolitical forces on neurosurgical training in sub-Saharan Africa. R Dempsey et al. World Neurosurg 101:196–202, 2017
9. Global Neurosurgery: the unmet need. Park KB, Johnson WD, Dempsey RJ. World Neurosurg. 2016;88:32–35
10. Towards a common definition of global health J P Koplan, TC Bond, MH Merson, K Srinath Reddy, MH Rodriguez, NK Sewankambo, JN Wasserheit, for the Consortium of Universities for Global Health Executive Board* Lancet 2009; 373: 1993–95

## Electronic supplementary material

ESM 1 (JPG 241 kb)

