# Supplementary figures and images for: Presidential address 2017 William Harkness FRCS October 10th 2017 Denver, Co USA: 2017—annus mirabilis, a global view of neurosurgery for children
Source: Childs Nerv Syst. 2018 Aug 18;34(10):1817–36. doi: 10.1007/s00381-018-3931-6 (PMC6133082; doi:10.1007/s00381-018-3931-6)

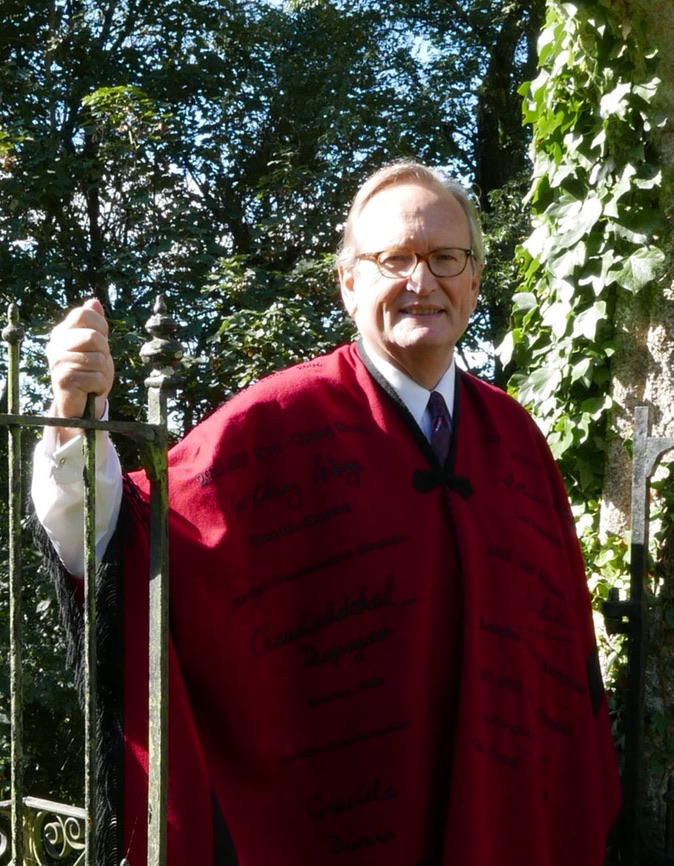

Supplement: Supplementary file 1 — (JPG 241 kb) [file 381_2018_3931_MOESM1_ESM.jpg]
